# National trends of metabolically healthy and unhealthy obesity before and during the COVID-19 pandemic, 2007 to 2021: A representative serial study in South Korea

**DOI:** 10.1097/MD.0000000000047797

**Published:** 2026-02-28

**Authors:** Ji Ho Kim, Yesol Yim, Hyunyi Yoo, Hyunjeong Kim, Selin Woo, Yerin Hwang, Dong Keon Yon

**Affiliations:** a Center for Digital Health, Medical Science Research Institute, Kyung Hee University Medical Center, Kyung Hee University College of Medicine, Seoul, South Korea; b Department of Precision Medicine, Kyung Hee University College of Medicine, Seoul, South Korea; c Department of Pediatrics, Kyung Hee University Medical Center, Kyung Hee University College of Medicine, Seoul, South Korea.

**Keywords:** metabolic syndrome, metabolically healthy obesity, metabolically unhealthy obesity, South Korea, trend

## Abstract

Long-term, nationally representative trends in metabolically healthy obesity (MHO) and metabolically unhealthy obesity (MUHO) in South Korea remain limited. Thus, we aimed to analyze the prevalence trends of MHO and MUHO and compare rates before and during the coronavirus disease 2019 (COVID-19) pandemic. We analyzed adults aged ≥ 19 years from the Korea National Health and Nutrition Examination Survey (2007–2021), a nationally representative repeated cross-sectional survey. Obese adults were classified as MHO or MUHO using National Cholesterol Education Program Adult Treatment Panel III (NCEP-ATP III) guidelines. To assess pandemic impact, data were divided into before (2007–2019) and during (2020–2021) the pandemic. National prevalence and temporal changes were estimated using weighted linear and logistic regression with 95% confidence intervals (CIs). A total of 87,625 adults were included (56.51% female). MUHO prevalence consistently exceeded MHO across all years. In 2021, MHO prevalence was 3.63% (95% CI = 2.97–4.28) versus 15.02% (13.05–16.99) for MUHO. Before the pandemic, MHO prevalence increased steadily (β = 0.035 [95% CI = 0.016–0.055]) and continued to rise during the pandemic (β = 0.055 [0.006–0.104]). MUHO prevalence, however, grew more sharply before the pandemic (β = 0.073 [0.036–0.111]) but plateaued during the pandemic (β = 0.061 [–0.031 to 0.153]). This nationwide cross-sectional study highlights distinct trajectories of MHO and MUHO before and during COVID-19. Given the higher and persistent prevalence of MUHO, personalized strategies tailored to metabolic health status are essential in managing obesity in Korea, with important implications for public health policy development.

## 1. Introduction

Obesity has been recognized as a major global health concern owing to its steadily increasing prevalence over recent decades,^[1]^ and its serious complications along with a strong association with metabolic syndrome and consequent cardiovascular diseases.^[2]^ However, a subset of patients with obesity maintains a healthy metabolic profile despite their excessive adiposity.^[3]^ These individuals display none of the traditional cardiometabolic risk markers – hypertension, impaired glucose regulation, or dyslipidemia – commonly observed with elevated body fat. Therefore, the distinction between metabolically healthy obesity (MHO) and metabolically unhealthy obesity (MUHO) – which reflects the more nuanced spectrum of obesity phenotypes – has become increasingly important.^[4]^

In this context, studies in several countries have investigated various metabolic diseases,^[5]^ physical performance,^[6]^ and related conditions using the MHO and MUHO phenotypes.^[7]^ These studies have highlighted the need for a more robust and universally accepted definition of obesity phenotypes to enable better stratification of individuals with obesity and facilitate health managements.^[8]^ Thus, categorizing individuals with obesity into MHO and MUHO using relatively stringent criteria and analyzing long-term trends is essential not only for accurately assessing the health status of the obese population but also for addressing and supporting efforts to reduce health disparities among individuals.^[6]^ Although many studies in South Korea have investigated the associations between MHO and MUHO with various cardiometabolic conditions,^[9,10]^ evidence on temporal trends in their prevalence among adults remains limited.^[11]^ To address this gap, we examined long-term trends in MHO and MUHO among Korean adults from 2007 to 2021. Furthermore, we examined these trends before and during the coronavirus disease 2019 (COVID-19) pandemic to evaluate the pandemic’s potential impact on changes in the prevalence of MHO and MUHO. Analyzing these trends among Korean adults is expected to provide valuable insights for accurately classifying diverse health conditions – especially among individuals with obesity – and for addressing health disparities.

## 2. Methods

### 2.1. Data source

This study used data from the Korea National Health and Nutrition Examination Survey (KNHANES), an annual survey conducted by the Korea Disease Control and Prevention Agency from 2007 to 2021.^[12]^ The present study employed sampling weights to ensure that the collected data accurately represented the distribution of the South Korean population, thereby enabling a precise evaluation of national health and nutritional status.^[13]^ Among the 128,331 individuals initially surveyed, 27,830 were excluded due to being under 19 years of age, resulting in 100,501 adults. For the complete case analysis,^[5]^ participants with any missing values were further excluded: 6119 had missing education level data, 3966 had missing high-density lipoprotein cholesterol (HDL-C) data, 1652 had missing blood pressure data, 1024 had missing household income data, 525 had missing body mass index (BMI) data, 12 had missing smoking status data, and 3 had missing sampling weights. In total, 12,876 individuals were excluded due to missing data, resulting in a final study population of 87,625 adults for analyzing trends in MHO and MUHO. The number of participants was grouped according to survey period as follows: 16,079 participants in 2007 to 2009; 16,711 in 2010 to 2012; 26,008 in 2013 to 2016; 17,884 in 2017 to 2019; 5601 in 2020; and 5342 in 2021 (Fig. S1, Supplemental Digital Content, https://links.lww.com/MD/R453). The study was approved by the Institutional Review Board of the Korea Disease Control and Prevention Agency (2007-02CON-04-P, 2008-04EXP-01-C, 2009-01CON-03-2C, 2010-02CON-21-C, 2011-02CON-06-C, 2012-01EXP-01-2C, 2013-07CON-03-4C, 2013-12EXP-03-5C, 2018-01-03-P-A, 2018-01-03-C-A, 2018-01-03-2C-A, 2018-01-03-5C-A, and 2018-01-03-4C-A), and written informed consent was obtained from all participants. The study was conducted using the principles outlined in the Declaration of Helsinki.

### 2.2. Definition of MHO and MUHO

We classified adults with obesity into MHO and MUHO phenotypes using metabolic health criteria from the National Cholesterol Education Program Adult Treatment Panel III (NCEP-ATP III) guidelines for metabolic syndrome.^[14]^ These criteria are validated and widely applied in Asian populations.^[10,15]^ To define metabolic health status, we assessed 5 metabolic abnormalities^[5,9]^: 1) elevated blood pressure: systolic blood pressure ≥ 130 mm Hg, diastolic blood pressure ≥ 85 mm Hg, or current use of antihypertensive medication; 2) impaired glucose metabolism: fasting blood glucose ≥ 110 mg/dL or non-fasting blood glucose ≥ 126 mg/dL, current use of blood glucose-lowering medication, or physician-diagnosed type 2 diabetes; 3) reduced HDL-C: <40 mg/dL for males and < 50 mg/dL for females, or medical treatment for low HDL-C; 4) elevated triglycerides (TG): ≥150 mg/dL or medication for elevated TG; or 5) cardiovascular disease history: physician-diagnosed angina, myocardial infarction, or stroke.^[16]^ Following established approaches, we defined MHO as obesity (BMI ≥ 25 kg/m^2^) with none of the 5 metabolic abnormalities. MUHO was defined as obesity with at least 1 metabolic abnormality.^[9,17]^

### 2.3. Covariates

We considered 9 covariates related to participant characteristics for the MHO and MUHO analysis: sex (male and female), age groups (19–39, 40–59, and ≥ 60 years),^[18]^ region of residence (urban and rural),^[19]^ and obesity class as defined by the Korean Society for the Study of Obesity guidelines (class I [BMI, 25.0–29.9 kg/m^2^], class II [BMI, 30.0–34.9 kg/m^2^], and class III [BMI ≥ 35.0 kg/m^2^]).^[20]^ Additional covariates included drinking status (defined as drinking at least once a month within the past year), binge drinking status (defined as consuming an average of 7 or more drinks per occasion for male, 5 or more for female, and drinking at least twice a week),^[21]^ smoking status (defined as individuals who are current or occasional smokers), educational background (high school or lower, and college or higher), and household income level (lowest and second quartile, and third and highest quartile).^[22]^

### 2.4. Statistical analyses

We examined trends in the prevalence of MHO and MUHO using data from the KNHANES grouped by year from 2007 to 2021. The survey period was categorized into the following time periods: 2007 to 2009, 2010 to 2012, 2013 to 2016, 2017 to 2019, 2020, and 2021. The years 2020 and 2021 were considered separately as pandemic years, as the first case of COVID-19 in South Korea was reported on January 20, 2020.^[15,23]^ Study results are presented as ratios or percentages derived from quantitative data. To ensure precise estimates, a weighted composite sampling analysis was conducted to account for differences in the number of participants across survey years.^[13]^ For statistical analysis, we used weighted linear regression and weighted binomial logistic regression models to calculate β-coefficients and weighted odds ratios (wORs), respectively, each with a 95% confidence intervals (CIs). To enhance the reliability of the finding, stratified analyses were conducted, incorporating variables such as sex, age, region of residence, obesity class, alcohol consumption, binge drinking, smoking status, education level, and household income as covariates in all regression models. All statistical analyses were performed SAS software (version 9.4; SAS Institute, Cary, NC, USA), and all tests were two-sided, with statistical significance defined as a *P*-value < 0.05.

## 3. Results

Among the 100,501 participants initially surveyed in the KNHANES between 2007 and 2021, a total of 87,625 were included in the analysis after excluding 12,876 individuals with missing data on education level, smoking status, household income, BMI, HDL‑C, blood pressure, or sampling weights (Fig. S1, Supplemental Digital Content, https://links.lww.com/MD/R453). Other weighted sociodemographic characteristics are summarized in Table 1.

**Table 1:** Baseline characteristics of Korean based on data obtained from the KNHANES, 2007 to 2021 (n = 87,625).

Characteristic	Total	Before the pandemic				During the pandemic	
		2007–2009	2010–2012	2013–2016	2017–2019	2020	2021
Overall	87,625	16,079	16,711	26,008	17,884	5601	5342
Crude rate, n (%)							
Sex, n (%)							
Male	38,108 (43.49)	6872 (7.84)	7143 (8.15)	11,257 (12.85)	7957 (9.08)	2517 (2.87)	2362 (2.70)
Female	49,517 (56.51)	9207 (10.51)	9568 (10.92)	14,751 (16.83)	9927 (11.33)	3084 (3.52)	2980 (3.40)
Age group, n (%)							
19–39 yr	25,798 (29.44)	5475 (6.25)	5112 (5.83)	7607 (8.68)	4835 (5.52)	1511 (1.72)	1258 (1.44)
40–59 yr	32,848 (37.49)	5961 (7.19)	6298 (7.19)	9930 (11.33)	6784 (7.74)	1965 (2.24)	1910 (2.18)
≥60 yr	28,979 (33.07)	4643 (5.30)	5301 (6.05)	8471 (9.67)	6265 (7.15)	2125 (2.43)	2174 (2.48)
Region of residence, n (%)							
Urban	39,984 (45.63)	6927 (7.91)	7675 (8.77)	12,114 (13.84)	8299 (9.48)	2474 (2.83)	2475 (2.83)
Rural	47,641 (54.67)	9117 (10.41)	9008 (10.29)	13,891 (15.87)	9584 (10.95)	3127 (3.57)	2867 (3.27)
BMI group, n (%)*							
Non-obese	58,077 (66.28)	10,971 (12.52)	11,391 (13.00)	17,171 (19.60)	11,704 (13.36)	3453 (3.94)	3387 (3.87)
Obese	29,548 (33.72)	5108 (5.83)	5320 (6.07)	8837 (10.09)	6180 (7.05)	2148 (2.45)	1955 (2.23)
Obesity class (BMI), kg/m^2^ n (%)†							
Class I (25.0–29.9)	25,368 (28.95)	4509 (5.15)	4689 (5.35)	7559 (8.63)	5225 (5.96)	1768 (2.02)	1618 (1.85)
Class II (30.0–34.9)	3627 (4.14)	540 (0.62)	545 (0.62)	1124 (1.28)	809 (0.92)	321 (0.37)	288 (0.33)
Class III (≥35.0)	553 (0.63)	59 (0.07)	86 (0.10)	154 (0.18)	146 (0.17)	59 (0.07)	49 (0.06)
Drinking status, n (%)							
Yes	46,488 (53.05)	8556 (9.76)	8819 (10.06)	7701 (8.79)	15,935 (18.19)	2871 (3.28)	2606 (2.97)
No	41,137 (46.95)	7523 (8.59)	7892 (9.01)	6623 (7.56)	13,633 (15.56)	2730 (3.12)	2736 (3.12)
Binge drinking, n (%)							
Yes	16,914 (19.30)	1870 (2.13)	1763 (2.01)	2788 (3.18)	2087 (2.38)	630 (0.72)	565 (0.65)
No	70,711 (80.70)	14,209 (16.22)	14,948 (17.06)	23,220 (26.50)	15,797 (18.03)	4971 (5.67)	4777 (5.45)
Smoking status, n (%)							
Yes	16,903 (19.30)	3581 (4.09)	3449 (3.94)	4903 (5.60)	3210 (3.66)	927 (1.06)	844 (0.96)
No	70,655 (80.70)	12,498 (14.26)	13,262 (15.14)	21,105 (24.09)	14,674 (16.75)	4674 (5.33)	4498 (5.13)
Level of education, n (%)							
High school or lower education	50,474 (57.60)	10,746 (12.26)	10,425 (11.90)	14,639 (16.71)	9259 (10.57)	2675 (3.05)	2730 (3.12)
College or higher education	34,881 (39.81)	5301 (6.05)	6265 (7.15)	10,686 (12.20)	7790 (8.89)	2514 (2.87)	2325 (2.65)
Unknown	2270 (2.59)	32 (0.04)	21 (0.02)	683 (0.78)	835 (0.95)	412 (0.47)	287 (0.33)
Household income, n (%)							
Lowest and second quartile	38,561 (44.01)	7338 (8.37)	7429 (8.48)	11,356 (12.96)	7790 (8.89)	2341 (2.67)	2307 (2.63)
Third and highest quartile	49,064 (55.99)	8741 (9.98)	9282 (10.59)	14,652 (16.72)	10,094 (11.52)	3260 (3.72)	3035 (3.46)
Weighted rate (95% CI)							
Sex, weighted % (95% CI)							
Male	50.00 (49.30–50.38)	50.01 (49.28–50.75)	49.80 (49.01–50.59)	49.90 (49.22–50.58)	50.14 (49.40–50.88)	49.93 (48.82–51.05)	50.17 (48.83–51.52)
Female	50.00 (49.62–50.39)	49.99 (49.26–50.72)	50.20 (49.41–50.99)	50.10 (49.42–50.78)	49.86 (49.12–50.60)	50.07 (48.95–51.18)	49.83 (48.48–51.17)
Age, weighted % (95% CI)							
19–39 yr	37.09 (36.40–37.79)	43.14 (41.68–44.60)	40.59 (39.22–41.96)	37.59 (36.36–38.82)	35.32 (34.06–36.58)	34.12 (31.80–36.44)	33.21 (30.98–35.44)
40–59 yr	39.88 (39.30–40.46)	39.14 (37.89–40.38)	40.44 (39.32–41.55)	41.05 (40.12–42.07)	40.23 (39.13–41.32)	39.45 (37.32–41.59)	38.83 (37.08–40.58)
≥60 yr	23.03 (22.40–23.66)	17.73 (16.80–18.66)	18.98 (18.01–19.94)	21.37 (20.36–22.38)	24.45 (23.17–25.74)	26.42 (24.03–28.82)	27.96 (25.58–30.34)
BMI, weighted % (95% CI)*							
Not obese	65.10 (64.59–65.61)	68.26 (67.33–69.18)	67.77 (66.79–68.76)	66.19 (65.27–67.12)	65.27 (64.38–66.17)	61.13 (59.55–62.70)	62.78 (61.02–64.55)
Obese	34.90 (34.39–35.41)	31.74 (30.82–32.67)	32.23 (31.25–33.21)	33.81 (33.83–35.62)	34.72 (33.83–35.62)	38.87 (37.30–40.45)	37.22 (35.46–38.98)
Obesity class (BMI), kg/m^2^, weighted % (95% CI)†							
Class I (25.0–29.9)	29.28 (28.81–29.75)	27.82 (27.00–28.65)	27.76 (26.86–28.67)	28.74 (27.91–29.58)	29.15 (28.32–29.99)	31.59 (30.10–33.08)	30.19 (28.60–31.77)
Class II (30.0–34.9)	4.77 (4.52–5.02)	3.51 (3.16–3.86)	3.74 (3.31–4.16)	4.44 (4.08–4.81)	4.65 (4.23–5.06)	6.01 (5.18–6.83)	5.95 (5.05–6.84)
Class III (≥35.0)	0.86 (0.75–0.96)	0.41 (0.29–0.54)	0.73 (0.54–0.92)	0.63 (0.49–0.76)	0.93 (0.74–1.13)	1.28 (0.89–1.67)	1.08 (0.71–1.45)
Region of residence, weighted % (95% CI)							
Urban	46.50 (45.11–47.90)	47.02 (44.91–49.14)	46.84 (44.59–49.10)	47.18 (44.27–50.09)	46.64 (43.28–49.99)	45.37 (38.23–52.51)	46.02 (39.14–52.90)
Rural	53.50 (52.10–54.90)	52.98 (50.86–55.09)	53.16 (50.88–55.41)	52.82 (49.91–55.73)	53.36 (50.01–56.72)	54.63 (47.49–61.77)	53.98 (47.10–60.86)
Drinking status, weighted % (95% CI)							
Yes	57.40 (56.83–57.97)	59.08 (58.07–60.09)	58.87 (57.79–59.95)	58.85 (57.83–59.86)	58.63 (57.74–59.52)	55.61 (53.70–57.53)	53.85 (51.89–55.81)
No	42.60 (42.03–43.17)	40.92 (39.91–41.93)	41.13 (40.05–42.21)	41.15 (40.14–42.17)	41.37 (40.48–42.26)	44.39 (42.47–46.30)	46.15 (44.19–48.11)
Binge drinking, weighted % (95% CI)							
Yes	13.16 (12.80–13.52)	13.96 (13.23–14.69)	13.73 (12.99–14.48)	12.80 (12.13–13.46)	13.07 (12.80–13.52)	13.20 (12.07–14.34)	12.45 (11.30–13.60)
No	86.84 (86.48–87.20)	86.04 (85.31–86.77)	86.27 (85.52–87.01)	87.20 (86.54–87.87)	86.93 (86.29–87.58)	86.80 (85.66–87.93)	87.55 (86.40–88.70)
Smoking status, weighted % (95% CI)							
Yes	22.27 (21.80–22.74)	27.08 (26.21–27.96)	26.67 (25.72–27.62)	22.84 (21.95–23.73)	20.97 (20.10–21.84)	19.10 (17.61–20.59)	18.35 (16.90–19.75)
No	77.73 (77.26–78.20)	72.92 (72.04–73.79)	73.33 (72.39–74.28)	77.16 (76.27–78.05)	79.03 (78.16–79.90)	80.90 (79.41–82.40)	81.65 (80.25–83.10)
Level of education, weighted % (95% CI)							
High school or lower education	48.99 (48.13–49.87)	59.43 (57.88–60.98)	56.83 (55.30–58.36)	49.60 (48.08–51.12)	44.99 (43.32–46.66)	42.17 (39.18–45.16)	43.70 (40.82–46.59)
College or higher education	47.67 (46.77–48.56)	40.39 (38.84–41.94)	43.08 (41.56–44.30)	47.63 (46.07–49.19)	50.24 (48.51–51.97)	51.31 (48.08–54.54)	51.54 (48.47–54.61)
Unknown	3.34 (3.05–3.62)	0.18 (0.11–0.26)	0.09 (0.04–0.14)	2.77 (2.37–3.17)	4.77 (4.21–5.33)	6.52 (5.27–7.76)	4.76 (3.79–5.73)
Household income, weighted % (95% CI)							
Lowest and second quartile	38.90 (37.95–39.85)	41.82 (38.94–42.50)	42.48 (40.91–44.06)	39.23 (37.51–40.95)	39.42 (37.66–41.17)	36.31 (32.85–39.77)	36.06 (32.79–39.34)
Third and highest quartile	61.10 (60.15–62.05)	59.25 (57.50–61.06)	57.42 (55.94–59.09)	60.77 (59.05–62.49)	60.58 (58.83–62.34)	63.69 (60.23–67.15)	63.94 (60.66–67.21)

BMI = body mass index, CI = confidence interval, KNHANES = Korea National Health and Nutrition Examination Survey.

* According to Asian-Pacific guidelines, BMI is divided into 4 groups: underweight (<18.5 kg/m^2^), normal (18.5–22.9 kg/m^2^), overweight (23.0–24.9 kg/m^2^), and obese (≥25.0 kg/m^2^).

† Obesity classes were defined based on BMI according to the Korean Society for the Study of Obesity guidelines: class I (25.0–29.9 kg/m^2^), class II (30.0–34.9 kg/m^2^), and class III (≥ 35.0 kg/m^2^).

**Figure 1.**
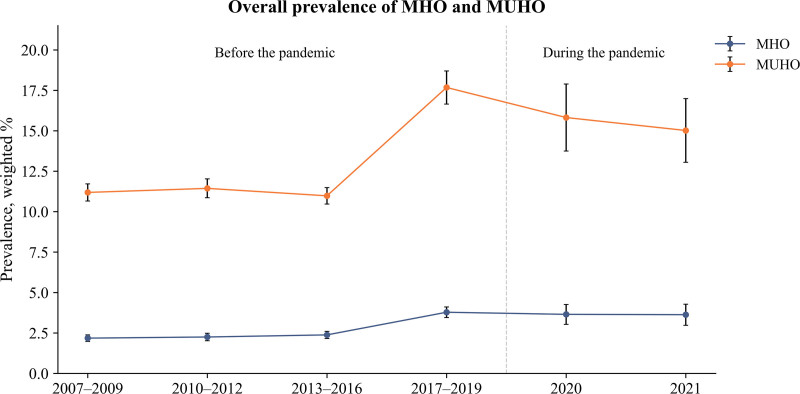
Nationwide trends in the prevalence of MHO and MUHO among 87,625 Korean adults, 2007 to 2021. Estimates are presented with error bars representing 95% confidence intervals. MHO = metabolically healthy obesity, MUHO = metabolically unhealthy obesity.

Figure 1 and Table 2 present the prevalence of MHO and MUHO from 2007 to 2021, comparing periods before and during the COVID-19 pandemic. Throughout the study period, MUHO prevalence consistently exceeded MHO prevalence, with MHO increasing from 2.18% (95% CI = 1.98–2.38) in 2007 and 2009 to 3.63% (95% CI = 2.97–4.28) in 2021, and MUHO rising from 11.19% (95% CI = 10.66–11.72) to 15.02% (95% CI = 13.05–16.99). When comparing periods before (2007–2019) and during (2020–2021) the pandemic, MHO prevalence increased steadily before the pandemic (β = 0.035 [95% CI = 0.016–0.055]) and continued to rise during the pandemic (β = 0.055 [95% CI = 0.006–0.104]). In contrast, MUHO prevalence increased more sharply before the pandemic (β = 0.073 [95% CI = 0.036–0.111]) but plateaued during the pandemic period (β = 0.061 [95% CI = −0.031 to 0.153]).

**Table 2:** Trends in the prevalence of metabolic healthy and β-coefficients before and during the COVID-19 pandemic (weighted % [95% CI]) among metabolically healthy obesity and metabolically unhealthy obesity group based on data obtained from the KNHANES.

Variables	Group	Before the pandemic				During the pandemic		Trends in the before pandemic, β (95% CI)	Trends in the pandemic, β (95% CI)	β_diff_ between 2007–2019 and 2019–2021 (95% CI)
		2007–2009	2010–2012	2013–2016	2017–2019	2020	2021			
Overall	MHO	2.18 (1.98–2.38)	2.25 (2.02–2.48)	2.38 (2.16–2.60)	3.78 (3.45–4.11)	3.65 (3.03–4.26)	3.63 (2.97–4.28)	**0.035 (0.016–0.055**)***	0.055 (0.006–0.104)*	0.019 (−0.034 to 0.072)
	MUHO	11.19 (10.66–11.72)	11.44 (10.86–12.03)	10.98 (10.47–11.49)	17.68 (16.65–18.70)	15.82 (13.75–17.89)	15.02 (13.05–16.99)	**0.073 (0.036–0.111**)***	0.061 (−0.031 to 0.153)	−0.012 (−0.111 to 0.086)
Sex										
Male	MHO	2.16 (1.89–2.42)	2.09 (1.79–2.40)	2.37 (2.07–2.66)	3.73 (3.34–4.13)	3.83 (3.06–4.59)	3.56 (2.86–4.25)	**0.044 (0.014–0.075**)**	0.062 (−0.007 to 0.132)	0.018 (−0.058 to 0.094)
	MUHO	10.79 (10.18–11.41)	10.94 (10.26–11.61)	10.89 (10.28–11.50)	17.94 (16.79–19.09)	16.34 (14.12–18.55)	15.37 (13.17–17.57)	**0.145 (0.090–0.200**)***	0.089 (−0.042 to 0.220)	−0.056 (−0.198 to 0.086)
Female	MHO	2.21 (1.94–2.47)	2.48 (2.17–2.80)	2.41 (2.14–2.68)	3.85 (3.43–4.26)	3.39 (2.69–4.08)	3.73 (2.75–4.71)	0.026 (0.004–0.049)*	0.047 (−0.023 to 0.118)	0.021 (−0.053 to 0.095)
	MUHO	11.76 (11.07–12.45)	12.17 (11.44–12.90)	11.12 (10.46–11.77)	17.30 (16.15–18.45)	15.09 (12.91–17.26)	14.51 (12.49–16.52)	0.001 (−0.047 to 0.048)	0.032 (−0.086 to 0.151)	0.032 (−0.096 to 0.159)
Age group										
19–39 yr	MHO	3.66 (3.18–4.13)	3.81 (3.30–4.33)	4.15 (3.62–4.68)	6.25 (5.58–6.91)	6.64 (5.37–7.91)	6.09 (4.73–7.45)	**0.095 (0.058–0.132**)***	0.139 (0.028–0.250)*	0.044 (−0.074 to 0.161)
	MUHO	10.65 (9.81–11.48)	10.59 (9.63–11.56)	9.27 (8.46–10.09)	14.80 (13.56–16.04)	12.82 (10.68–14.96)	11.27 (9.24–13.30)	0.085 (0.032–0.139)	0.010 (−0.139 to 0.160)	−0.075 (−0.234 to 0.084)
40–59 yr	MHO	1.94 (1.68–2.20)	2.03 (1.74–2.33)	1.93 (1.66–2.20)	3.41 (2.98–3.85)	2.63 (2.04–3.22)	3.15 (2.42–3.88)	0.027 (−0.002 to 0.056)	0.049 (−0.024 to 0.123)	0.022 (−0.057 to 0.101)
	MUHO	12.18 (11.38–12.98)	12.34 (11.52–13.16)	11.85 (11.09–12.62)	17.90 (16.64–19.16)	15.78 (13.35–18.21)	14.86 (12.53–17.18)	**−0.023 (−0.082 to 0.035**)**	0.098 (−0.046 to 0.241)	0.121 (−0.034 to 0.276)
≥60 yr	MHO	0.63 (0.48–0.78)	0.57 (0.42–0.71)	0.83 (0.64–1.02)	1.16 (0.94–1.39)	1.46 (1.00–1.91)	1.20 (0.69–1.71)	0.001 (−0.018 to 0.020)	0.008 (−0.039 to 0.054)	0.006 (−0.044 to 0.056)
	MUHO	10.19 (9.43–10.96)	11.01 (10.19–11.84)	11.73 (10.92–12.55)	21.10 (19.54–22.67)	19.87 (16.98–22.75)	20.24 (17.35–23.14)	0.076 (0.008–0.144)*	−0.004 (−0.142 to 0.134)	−0.081 (−0.234 to 0.073)
Region of residence										
Urban	MHO	2.29 (1.98–2.59)	2.55 (2.15–2.95)	2.26 (1.91–2.60)	3.97 (3.46–4.47)	3.54 (2.65–4.44)	4.28 (3.26–5.31)	0.028 (−0.001 to 0.056)	**0.105 (0.032–0.178**)**	0.078 (−0.001 to 0.156)
	MUHO	11.59 (10.69–12.49)	11.00 (10.11–11.90)	11.03 (10.21–11.85)	17.29 (15.78–18.81)	15.57 (12.51–18.63)	14.63 (11.78–17.47)	0.037 (−0.016 to 0.090)	0.081 (−0.043 to 0.206)	0.044 (−0.091 to 0.179)
Rural	MHO	2.09 (1.81–2.37)	2.01 (1.73–2.29)	2.48 (2.18–2.79)	3.63 (3.18–4.07)	3.73 (2.89–4.58)	3.10 (2.24–3.95)	**0.042 (0.015–0.070**)**	0.012 (−0.055 to 0.078)	−0.031 (−0.103 to 0.041)
	MUHO	10.86 (10.11–11.62)	11.80 (10.92–12.68)	10.94 (10.17–11.72)	17.99 (16.50–19.49)	16.03 (13.20–18.87)	15.33 (12.58–18.08)	**0.105 (0.053–0.157**)***	0.041 (−0.091 to 0.172)	−0.064 (−0.206 to 0.077)
Obesity class†										
Class I	MHO	2.41 (2.19–2.64)	2.42 (2.16–2.68)	2.57 (2.32–2.82)	4.05 (3.69–4.41)	3.82 (3.16–4.48)	3.82 (3.12–4.53)	0.060 (0.001–0.120)*	0.121 (−0.017 to 0.259)	0.061 (−0.090 to 0.211)
	MUHO	11.54 (10.99–12.10)	11.63 (11.03–12.24)	11.04 (10.50–11.58)	17.47 (16.44–18.49)	15.03 (13.01–17.04)	14.19 (12.30–16.08)		−0.121 (−0.259 to 0.017)	−0.061 (−0.211 to 0.090)
Class II	MHO	1.10 (0.70–1.50)	1.35 (0.87–1.84)	1.57 (1.12–2.01)	2.46 (1.86–3.07)	2.45 (1.44–3.45)	2.82 (1.49–4.16)	0.043 (−0.096 to 0.182)	0.064 (−0.246 to 0.375)	0.021 (−0.319 to 0.362)
	MUHO	9.73 (8.62–10.84)	10.28 (9.01–11.55)	10.94 (9.72–12.16)	18.68 (16.79–20.57)	19.60 (16.25–22.95)	19.01 (15.56–22.47)	−0.043 (−0.182 to 0.096)	−0.064 (−0.375 to 0.246)	−0.021 (−0.362 to 0.319)
Class III	MHO	0.07 (0.00–0.21)	1.43 (0.41–2.45)	0.42 (0.00–1.02)	1.86 (0.72–3.00)	4.38 (0.92–7.83)	1.31 (0.00–2.81)	0.118 (−0.128 to 0.364)	−0.157 (−0.583 to 0.268)	−0.275 (−0.767 to 0.216)
	MUHO	7.09 (4.84–9.33)	11.39 (8.16–14.62)	9.34 (6.91–11.77)	19.38 (15.34–23.42)	22.19 (15.62–28.75)	21.15 (14.25–28.06)	−0.118 (−0.364 to 0.128)	0.157 (−0.268 to 0.583)	0.275 (−0.216 to 0.767)
Drinking status										
Yes	MHO	2.45 (2.17–2.74)	2.59 (2.26–2.91)	2.56 (2.26–2.85)	4.23 (3.80–4.65)	4.20 (3.44–4.95)	3.68 (2.93–4.43)	−0.045 (−0.120 to 0.030)	−0.091 (−0.254 to 0.073)	−0.046 (−0.226 to 0.134)
	MUHO	11.14 (10.50–11.77)	11.54 (10.82–12.26)	10.99 (10.37–11.62)	17.62 (16.51–18.74)	15.22 (13.11–17.33)	13.79 (11.80–15.78)	−0.045 (−0.120 to 0.030)	−0.091 (−0.254 to 0.073)	−0.046 (−0.226 to 0.134)
No	MHO	1.79 (1.52–2.05)	1.78 (1.51–2.06)	2.13 (1.85–2.42)	3.15 (2.77–3.53)	2.87 (2.23–3.52)	3.55 (2.68–4.43)	−0.060 (−0.135 to 0.015)	−0.121 (−0.305 to 0.062)	−0.061 (−0.259 to 0.137)
	MUHO	11.26 (10.56–11.97)	11.31 (10.59–12.03)	10.97 (10.27–11.66)	17.75 (16.53–18.98)	16.68 (14.31–19.04)	16.75 (14.43–19.07)	−0.060 (−0.135 to 0.015)	−0.121 (−0.305 to 0.062)	−0.061 (−0.259 to 0.137)
Binge drinking										
Yes	MHO	2.22 (1.73–2.72)	2.18 (1.60–2.76)	1.80 (1.35–2.26)	2.87 (2.33–3.42)	3.51 (2.26–4.75)	2.39 (1.49–3.29)	−0.012 (−0.066 to 0.041)	0.030 (−0.093 to 0.153)	0.043 (−0.092 to 0.177)
	MUHO	12.12 (10.98–13.26)	12.49 (11.16–13.82)	11.56 (10.38–12.74)	18.02 (16.40–19.64)	16.41 (13.63–19.19)	14.42 (11.76–17.08)	0.104 (−0.005 to 0.212)	0.064 (−0.173 to 0.302)	−0.039 (−0.300 to 0.222)
No	MHO	2.17 (1.96–2.38)	2.27 (2.03–2.50)	2.50 (2.26–2.73)	3.96 (3.60–4.32)	3.67 (3.04–4.31)	3.87 (3.14–4.60)	**0.043 (0.022–0.064**)***	0.058 (0.004–0.113)*	0.015 (−0.043 to 0.074)
	MUHO	11.01 (10.46–11.56)	11.24 (10.66–11.82)	10.87 (10.33–11.41)	17.61 (16.57–18.66)	15.71 (13.61–17.81)	15.13 (13.13–17.14)	**0.072 (0.034–0.111**)***	0.064 (−0.032 to 0.159)	−0.009 (−0.112 to 0.094)
Smoking status										
Yes	MHO	2.66 (2.20–3.13)	2.36 (1.88–2.84)	2.52 (2.07–2.97)	3.36 (2.83–3.88)	3.27 (2.29–4.25)	2.83 (1.99–3.67)	**0.028 (−0.015 to 0.071**)***	0.076 (−0.039 to 0.190)	0.048 (−0.074 to 0.170)
	MUHO	13.50 (12.50–14.51)	13.62 (12.46–14.77)	12.05 (11.10–13.00)	17.61 (16.27–18.95)	13.56 (11.35–15.76)	12.66 (10.45–14.87)	**0.128 (0.050–0.206**)**	0.066 (−0.127 to 0.259)	−0.062 (−0.270 to 0.146)
No	MHO	2.02 (1.80–2.23)	2.22 (1.96–2.47)	2.34 (2.10–2.58)	3.92 (3.56–4.28)	3.77 (3.10–4.44)	3.89 (3.14–4.64)	0.039 (0.018–0.060)	0.051 (−0.003 to 0.105)	0.012 (−0.046 to 0.070)
	MUHO	10.43 (9.88–10.98)	10.73 (10.16–11.30)	10.63 (10.08–11.19)	17.70 (16.60–18.80)	16.57 (14.36–18.78)	15.79 (13.70–17.88)	**0.068 (0.027–0.110**)**	0.070 (−0.034 to 0.173)	0.001 (−0.110 to 0.113)
Education level										
High school or lower education	MHO	2.15 (1.89–2.41)	1.97 (1.72–2.21)	1.87 (1.64–2.11)	2.47 (2.17–2.77)	2.25 (1.73–2.77)	2.01 (1.51–2.51)	0.007 (−0.016 to 0.030)	0.013 (−0.044 to 0.069)	0.006 (−0.055 to 0.067)
	MUHO	14.24 (13.48–15.01)	14.11 (13.30–14.92)	12.46 (11.79–13.14)	17.63 (16.50–18.77)	13.83 (11.81–15.85)	15.00 (12.91–17.09)	**0.098 (0.050–0.147**)***	0.148 (0.028–0.267)*	0.049 (−0.080 to 0.179)
College or higher education	MHO	2.37 (2.06–2.69)	2.77 (2.34–3.19)	3.11 (2.72–3.50)	5.26 (4.71–5.80)	5.25 (4.26–6.25)	5.50 (4.38–6.62)	**0.050 (0.018–0.082**)**	0.087 (0.012–0.162)*	0.037 (−0.045 to 0.119)
	MUHO	8.34 (7.66–9.02)	9.12 (8.34–9.89)	9.71 (8.94–10.47)	17.00 (15.68–18.32)	17.04 (14.44–19.64)	14.53 (12.18–16.89)	**0.107 (0.055–0.159**)***	0.011 (−0.106 to 0.128)	−0.096 (−0.224 to 0.031)
Household income										
Lowest and second quartile	MHO	1.93 (1.63–2.23)	2.02 (1.70–2.35)	2.03 (1.73–2.33)	3.21 (2.80–3.62)	2.55 (1.98–3.12)	2.56 (1.92–3.20)	0.031 (0.002–0.059)*	0.018 (−0.048 to 0.084)	−0.013 (−0.085 to 0.060)
	MUHO	12.30 (11.46–13.14)	12.70 (11.85–13.55)	11.79 (11.02–12.57)	18.46 (17.17–19.75)	15.45 (13.14–17.75)	14.99 (12.71–17.27)	0.073 (0.014–0.133)*	0.140 (−0.001 to 0.281)	0.067 (−0.086 to 0.219)
Third and highest quartile	MHO	2.34 (2.07–2.62)	2.41 (2.09–2.72)	2.62 (2.32–2.92)	4.17 (3.72–4.61)	4.38 (3.58–5.19)	4.35 (3.46–5.23)	**0.037 (0.010–0.064**)**	0.071 (0.009–0.134)*	0.034 (−0.034 to 0.102)
	MUHO	10.44 (9.74–11.14)	10.59 (9.86–11.33)	10.43 (9.75–11.12)	17.15 (15.92–18.38)	16.08 (13.64–18.52)	15.04 (12.85–17.22)	**0.078 (0.031–0.124**)**	0.025 (−0.084 to 0.133)	−0.053 (−0.171 to 0.065)

BMI = body mass index, CI = confidence interval, MHO = metabolically healthy obese, MUHO = metabolically unhealthy obese, KNHANES = Korea National Health and Nutrition Examination Survey.

The beta values were multiplied by 10 owing to their minimal number.

The figures in bold represent a significant variance.

† Obesity classes were defined based on BMI according to the Korean Society for the Study of Obesity guidelines: class I (25.0–29.9 kg/m^2^), class II (30.0–34.9 kg/m^2^), and class III (≥ 35.0 kg/m^2^).

* *P*-value between 0.05 and 0.01.

** *P*-value between 0.01 and 0.001.

*** *P*-value <0.001.

**Figure 2.**
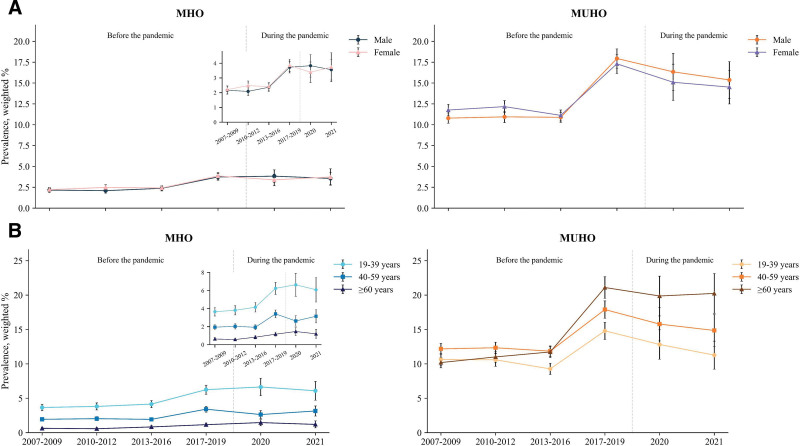
Nationwide trends in prevalence of MHO and MUHO among 87,625 Korean adults, by sex (A) and age group (B), 2007 to 2021. Estimates are presented with error bars representing 95% confidence intervals. For groups whose trends are not clearly visible due to y-axis scaling, inset figures are provided. MHO = metabolically healthy obesity, MUHO = metabolically unhealthy obesity.

Figure 2 presents sex- and age-group-specific prevalence rates of MHO and MUHO for periods before and during the COVID-19 pandemic. In sex-specific analyses, both males and females in the MHO group showed similar upward trends, increasing from approximately 2.2% in 2007 to 2009 to 3.6 to 3.7% in 2021. In contrast, the MUHO group exhibited a notable shift in sex patterns: females initially had higher prevalence than males in 2007 to 2009 (11.76% [95% CI = 11.07–12.45] vs 10.79% [95% CI = 10.18–11.41]), but males overtook females by 2017 to 2019 (17.94% [95% CI = 16.79–19.09] vs 17.30% [95% CI = 16.15–18.45]) and remained higher through 2021 (15.37% [95% CI = 13.17–17.57] vs 14.51% [95% CI = 12.49–16.52]). In age-specific analyses, MHO prevalence was consistently highest among individuals aged 19 to 39 years throughout all study periods, increasing from 3.66% (95% CI = 3.18–4.13) in 2007 to 2009 to 6.09% (95% CI = 4.73–7.45) in 2021. For MUHO, individuals aged ≥ 60 years showed a dramatic shift: from the lowest prevalence in 2007 to 2009 (10.19% [95% CI = 9.43–10.96]) to the highest by 2017 to 2019 (21.10% [95% CI = 19.54–22.67]), a pattern that persisted through 2021 (20.24% [95% CI = 17.35–23.14]). Further details on prevalence trends are provided in Table 2 and Table S1 (Supplemental Digital Content, https://links.lww.com/MD/R453).

**Figure 3.**
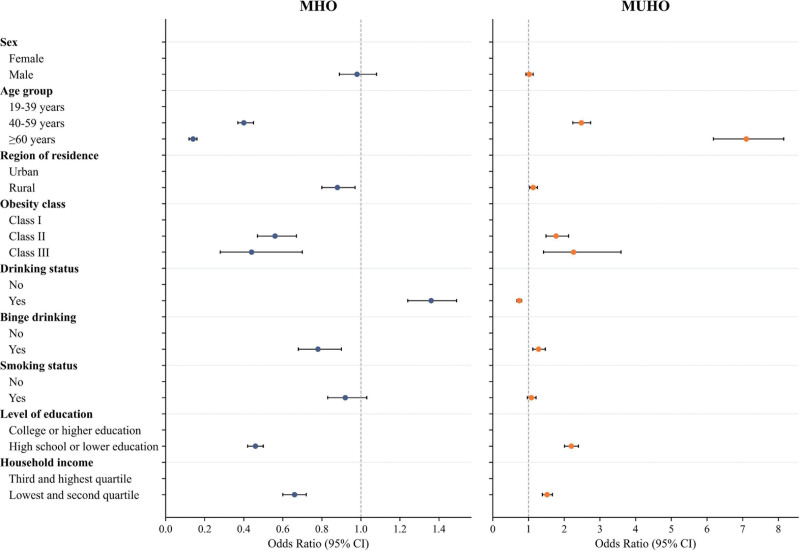
Weighted odds ratios for demographic and clinical characteristics according to MHO and MUHO status among Korean adults, 2007 to 2021. Estimates are presented with error bars representing 95% confidence intervals. Reference category: female, aged 19 to 39 years, urban residence, obesity class I, non‑drinkers, no binge drinking, non‑smokers, college‑level education or higher, and third and highest quartile household income. MHO = metabolically healthy obesity, MUHO = metabolically unhealthy obesity.

Figure 3 and Table S2 (Supplemental Digital Content, https://links.lww.com/MD/R453) present wORs for risk factors associated with MHO and MUHO. Risk factor patterns differed notably between the 2 phenotypes. MUHO was strongly associated with older age, particularly among those aged ≥ 60 years (wOR = 7.10 [95% CI = 6.18–8.15]) and aged 40 to 59 years (wOR = 2.48 [95% CI = 2.24–2.74]). Additional factors associated with higher MUHO prevalence included lower socioeconomic status, reflected in education level of high school or less (wOR = 2.20 [95% CI = 2.01–2.40]) and lowest income quartile (wOR = 1.52 [95% CI = 1.39–1.67]), as well as class III obesity (wOR = 2.26 [95% CI = 1.42–3.59]), binge drinking (wOR = 1.28 [95% CI = 1.12–1.47]), and rural residence (wOR = 1.13 [95% CI = 1.03–1.25]). In contrast, MHO showed the opposite pattern, being more prevalent among younger individuals with higher socioeconomic status.

## 4. Discussion

### 4.1. Key finding

This study presents a large‑scale analysis of long‑term trends in the prevalence of the MHO and MUHO groups over 15 years, from 2007 to 2021, involving 87,625 participants. Between 2007 to 2009 and 2021, MHO prevalence rose from 2.18 to 3.63%, while MUHO prevalence increased from 11.19 to 15.02%. Before the COVID-19 pandemic (2007–2019), both MHO and MUHO prevalence increased steadily, but during the pandemic period (2020–2021), MUHO prevalence plateaued while MHO prevalence continued to rise. Sex-specific analyses showed parallel increases in MHO prevalence among males and females, whereas MUHO prevalence was higher in females early in the study period but surpassed by males beginning in the 2017 to 2019 period. By age group, MHO prevalence was highest among adults aged 19 to 39 years, while MUHO prevalence shifted over time from being lowest in adults aged ≥60 years to highest in this group since 2017. MUHO was more prevalent among older adults, rural residents, individuals with class III obesity, binge drinkers, and those with lower levels of education or income, whereas MHO was more common in the complementary subgroups.

### 4.2. Plausible underlying mechanisms

The observed differences in prevalence and trends between MHO and MUHO may reflect the interplay of biological, behavioral, and sociodemographic factors. MUHO,^[24]^ characterized by the coexistence of obesity and metabolic abnormalities, is associated with chronic low-grade inflammation, insulin resistance, and endothelial dysfunction, all of which may be exacerbated in older adults, rural residents, and those with lower socioeconomic status due to reduced access to preventive healthcare and lifestyle interventions.^[25,26]^ In contrast, individuals with MHO may maintain more favorable metabolic profiles through healthier diet patterns, higher physical activity, and better health literacy despite elevated BMI.^[27]^ The temporal patterns observed in this study – continued increases in MHO prevalence during the pandemic alongside a plateau in MUHO – may have multiple explanations. Individuals with metabolic abnormalities likely received heightened medical attention during the pandemic,^[28,29]^ while lockdown conditions may have prompted health behavior improvements in this population.^[30]^ Limited access to restaurants, processed foods, and alcohol during restrictions could have further contributed to metabolic stabilization in the MUHO group.^[31]^

### 4.3. Comparison of previous studies

Our findings are consistent with prior population-based studies showing that MUHO is more prevalent than MHO and that MUHO disproportionately affects older adults, individuals with a high BMI, and socioeconomically disadvantaged groups.^[32–34]^ However, the observed shift in MUHO prevalence toward higher rates in females before 2017 to 2019 contrasts with earlier reports, which typically found a persistent male predominance.^[32,35]^ This divergence may reflect sex-specific differences in obesity management, occupational exposures, or hormonal and body composition changes over time. Furthermore, the reversal in age-related patterns, with adults aged ≥ 60 years exhibiting the highest MUHO prevalence in recent years, aligns with evidence of accelerated metabolic decline in older populations, particularly under reduced physical activity during the pandemic.^[36]^ Together, these results extend existing knowledge by capturing long-term trends across both pre- and post-pandemic periods in a large nationally representative cohort.

### 4.4. Clinical and policy implications

The different metabolic profiles and changing trends of MHO and MUHO populations have direct implications for health policy and clinical practice.^[37]^ First, obesity management needs a new approach. We recommend a 2-step screening system in national health programs: first measuring BMI, then requiring metabolic tests (blood sugar, cholesterol, blood pressure) for everyone with obesity. This approach helps identify who needs immediate help (MUHO) versus who needs preventive care (MHO). Given the elevated cardiovascular risk in MUHO individuals compared to healthy-weight people,^[37,38]^ early identification may help reduce disease burden.^[39]^ Second, our findings support age-tailored interventions. Older population (≥60 years) showed the highest MUHO burden, indicating the importance of routine metabolic screening in geriatric care.^[40]^ In contrast, younger adults (19–39 years), who exhibited predominantly MHO phenotypes, would benefit from workplace interventions that maintain metabolic health – shifting focus from traditional weight-loss programs to metabolic preservation strategies.^[41]^ Third, the differential pandemic impact on metabolic phenotypes has policy implications. MUHO rates plateaued during COVID-19 while MHO increased, possibly due to closer medical monitoring, lifestyle changes during lockdowns,^[42]^ or reduced access to unhealthy foods.^[31]^ These findings suggest that future emergency plans might benefit from prioritizing patients with MUHO for care, offering digital health interventions, and maintaining medication access.

### 4.5. Strength and limitations

A major strength of this study is its use of nationally representative data spanning 15 years (n = 87,625), which captures periods before and during the pandemic. By distinguishing obesity phenotypes into MHO and MUHO categories, the analysis offers a clearer basis for designing targeted interventions.

This study has several limitations. First, there is no universally accepted definition of MHO or MUHO; therefore, we applied commonly used NCEP-ATP III criteria.^[10,15]^ Second, the KNHANES data were collected via self-reported questionnaires, which may introduce subjective bias in variables such as drinking status, educational attainment, and income level. Moreover, the questionnaire variables only assessed angina, myocardial infarction, and stroke, which limited the ability to apply more comprehensive cardiovascular disease criteria. Nonetheless, the large sample size may mitigate some of these limitations.^[43]^ Third, missing data were handled using complete case analysis, which may have introduced selection bias by excluding participants with incomplete information. Although this approach is commonly used and was deemed appropriate for the present analysis, future studies may consider alternative methods such as multiple imputation to handle missing values more robustly and reduce potential bias.^[44]^ Fourth, the cross-sectional nature of this analysis allows for identification of associations but precludes any inference of causality. Longitudinal cohort studies are needed to examine transitions between MHO and MUHO phenotypes over time and identify modifiable factors that predict these transitions. Finally, this study was limited to the South Korean population, which may limit its applicability to other populations. Comparative studies across different populations and healthcare systems would clarify the generalizability of our findings and identify context-specific risk factors. However, our use of nationally representative data spanning 15 years provides important insights into metabolic obesity trends in Asian populations. These findings may serve as crucial evidence to inform the development of health policies tailored to these evolving trends.^[45]^

## 5. Conclusions

This 15-year nationwide analysis of more than 87,000 obese adults demonstrated that the prevalence of MUHO was consistently higher than that of MHO across all study periods and subgroups. The prevalence of MHO increased steadily over time, while that of MUHO plateaued during the COVID-19 pandemic. Notably, from 2017 to 2019, MUHO prevalence showed an upward trend among males and adults aged 60 years or older. Subgroup analyses indicated that MUHO was more common in older adults, rural residents, individuals with severe obesity, binge drinkers, and those with lower educational attainment and income levels. These findings underscore the heterogeneity within the obese population and the need for individualized clinical management and public health interventions tailored to distinct metabolic phenotypes. The divergent prevalence trends between the 2 groups, particularly during the pandemic, highlight the importance of continuous surveillance and targeted strategies to address metabolic health disparities during public health emergencies.

## Author contributions

**Data curation:** Ji Ho Kim.

**Methodology:** Yerin Hwang.

**Supervision:** Dong Keon Yon.

**Validation:** Yesol Yim.

**Visualization:** Yesol Yim.

**Writing – original draft:** Ji Ho Kim.

**Writing – review & editing:** Yesol Yim, Hyunyi Yoo, Hyunjeong Kim, Selin Woo, Yerin Hwang, Dong Keon Yon.

## Supplementary Material


