# Supplementary material for: National trends of metabolically healthy and unhealthy obesity before and during the COVID-19 pandemic, 2007 to 2021: A representative serial study in South Korea
Source: Medicine (Baltimore). 2026 Feb 28;105(9):e47797. doi: 10.1097/MD.0000000000047797 (PMC12956185; doi:10.1097/MD.0000000000047797)
Supplement: Supplementary file 1 [file medi-105-e47797-s001.pdf]

Supplementary Tables

Table S1. Temporal changes in metabolically healthy obesity and metabolically unhealthy obesity prevalence by sociodemographic characteristics across survey periods.

Variables	2010–2012 (reference) versus 2007–2009		2013–2016 (reference) versus 2010–2012		2017–2019 (reference) versus 2013–2016		2020 (reference) versus 2017–2019		2021 (reference) versus 2020	
	wOR (95% CI)	p-value	wOR (95% CI)	p-value	wOR (95% CI)	p-value	wOR (95% CI)	p-value	wOR (95% CI)	p-value
**MHO**										
**Overall**	1.01 (0.88 to 1.16)	0.878	1.10 (0.96 to 1.27)	0.180	0.99 (0.87 to 1.11)	0.819	1.08 (0.93 to 1.25)	0.323	1.05 (0.86 to 1.27)	0.637
**Sex**										
Male	0.96 (0.78 to 1.17)	0.673	1.14 (0.93 to 1.39)	0.222	0.96 (0.81 to 1.13)	0.611	1.13 (0.92 to 1.37)	0.241	0.99 (0.77 to 1.27)	0.926
Female	1.09 (0.91 to 1.30)	0.367	1.06 (0.89 to 1.27)	0.502	1.03 (0.87 to 1.21)	0.749	1.01 (0.81 to 1.26)	0.926	1.14 (0.83 to 1.57)	0.406
**Age**										
19–39 years	1.05 (0.85 to 1.29)	0.656	**1.24 (1.01 to 1.54)**	**0.044**	0.94 (0.79 to 1.13)	0.527	1.23 (0.99 to 1.52)	0.059	1.04 (0.77 to 1.42)	0.790
40–59 years	1.03 (0.84 to 1.27)	0.749	0.99 (0.80 to 1.22)	0.907	1.17 (0.97 to 1.42)	0.104	0.87 (0.70 to 1.09)	0.236	1.27 (0.95 to 1.70)	0.103
≥60 years	0.83 (0.58 to 1.18)	0.290	1.38 (0.97 to 1.95)	0.070	0.78 (0.58 to 1.05)	0.102	1.33 (0.93 to 1.91)	0.113	0.81 (0.48 to 1.35)	0.415
**Region of residence**										
Urban	1.17 (0.96 to 1.44)	0.124	0.88 (0.71 to 1.10)	0.258	1.12 (0.93 to 1.35)	0.225	0.99 (0.79 to 1.24)	0.940	1.29 (0.98 to 1.69)	0.072

Rural	0.89 (0.73 to 1.08)	0.232	**1.33 (1.10 to 1.61)**	**0.003**	0.89 (0.75 to 1.05)	0.155	1.15 (0.95 to 1.41)	0.154	0.87 (0.66 to 1.15)	0.320
**Obesity class***										
Class I	1.00 (0.86 to 1.15)	0.952	1.12 (0.96 to 1.30)	0.141	1.00 (0.88 to 1.13)	0.942	1.10 (0.94 to 1.28)	0.249	1.06 (0.86 to 1.30)	0.579
Class II	1.17 (0.69 to 1.98)	0.564	1.09 (0.68 to 1.75)	0.729	0.92 (0.62 to 1.37)	0.681	0.95 (0.58 to 1.55)	0.825	1.19 (0.62 to 2.29)	0.600
Class III	**12.53 (1.48 to 105.93)**	**0.020**	0.36 (0.07 to 1.89)	0.224	2.16 (0.43 to 10.91)	0.350	2.05 (0.70 to 5.98)	0.188	0.31 (0.07 to 1.39)	0.126
**Drinking status**										
Yes	1.02 (0.85 to 1.22)	0.856	1.04 (0.87 to 1.24)	0.680	1.03 (0.88 to 1.20)	0.697	1.15 (0.97 to 1.36)	0.102	0.97 (0.77 to 1.22)	0.778
No	0.99 (0.80 to 1.24)	0.945	1.24 (1.00 to 1.53)	0.053	0.91 (0.76 to 1.10)	0.327	0.97 (0.76 to 1.24)	0.811	1.23 (0.90 to 1.69)	0.193
**Binge drinking**										
Yes	0.95 (0.66 to 1.39)	0.800	0.89 (0.60 to 1.33)	0.575	1.02 (0.73 to 1.44)	0.895	1.34 (0.90 to 2.00)	0.155	0.78 (0.45 to 1.33)	0.356
No	1.02 (0.89 to 1.18)	0.759	1.14 (0.99 to 1.32)	0.080	0.98 (0.86 to 1.11)	0.744	1.04 (0.89 to 1.22)	0.628	1.09 (0.88 to 1.35)	0.416
**Smoking status**										
Yes	0.88 (0.66 to 1.18)	0.382	1.21 (0.90 to 1.62)	0.214	0.91 (0.71 to 1.17)	0.472	1.27 (0.91 to 1.76)	0.163	0.93 (0.59 to 1.45)	0.738
No	1.07 (0.91 to 1.25)	0.423	1.06 (0.91 to 1.25)	0.441	1.01 (0.88 to 1.15)	0.915	1.03 (0.87 to 1.22)	0.753	1.08 (0.87 to 1.35)	0.477
**Level of education**										
High school or lower education	0.92 (0.77 to 1.11)	0.386	1.08 (0.90 to 1.30)	0.424	0.93 (0.78 to 1.11)	0.430	1.16 (0.91 to 1.48)	0.224	0.82 (0.60 to 1.13)	0.225

College or higher education	1.07 (0.86 to 1.32)	0.562	1.06 (0.86 to 1.30)	0.611	0.97 (0.82 to 1.14)	0.670	1.00 (0.83 to 1.20)	0.977	1.23 (0.97 to 1.56)	0.092
**Household income**										
Lowest and second quartile	1.02 (0.81 to 1.27)	0.899	1.08 (0.86 to 1.35)	0.503	1.01 (0.83 to 1.23)	0.911	0.95 (0.74 to 1.22)	0.688	1.03 (0.75 to 1.43)	0.847
Third and highest quartile	1.01 (0.85 to 1.21)	0.900	1.11 (0.93 to 1.32)	0.258	0.97 (0.83 to 1.13)	0.666	1.12 (0.94 to 1.34)	0.205	1.06 (0.85 to 1.32)	0.599
**MUHO**										
**Overall**	0.99 (0.86 to 1.14)	0.878	0.91 (0.79 to 1.05)	0.180	1.01 (0.90 to 1.15)	0.819	0.93 (0.80 to 1.08)	0.323	0.95 (0.79 to 1.16)	0.637
**Sex**										
Male	1.05 (0.85 to 1.28)	0.673	0.88 (0.72 to 1.08)	0.222	1.04 (0.89 to 1.23)	0.611	0.89 (0.73 to 1.08)	0.241	1.01 (0.79 to 1.30)	0.926
Female	0.92 (0.77 to 1.10)	0.367	0.94 (0.79 to 1.12)	0.502	0.97 (0.83 to 1.15)	0.749	0.99 (0.79 to 1.23)	0.926	0.87 (0.64 to 1.20)	0.406
**Age**										
19–39 years	0.95 (0.78 to 1.17)	0.656	**0.80 (0.65 to 0.99)**	**0.044**	1.06 (0.88 to 1.27)	0.527	0.82 (0.66 to 1.01)	0.059	0.96 (0.70 to 1.31)	0.790
40–59 years	0.97 (0.79 to 1.19)	0.749	1.01 (0.82 to 1.25)	0.907	0.85 (0.71 to 1.03)	0.104	1.15 (0.92 to 1.43)	0.236	0.79 (0.59 to 1.05)	0.103
≥60 years	1.21 (0.85 to 1.72)	0.290	0.73 (0.51 to 1.03)	0.070	1.29 (0.95 to 1.74)	0.102	0.75 (0.53 to 1.07)	0.113	1.24 (0.74 to 2.07)	0.415
**Region of residence**										
Urban	0.85 (0.70 to 1.05)	0.124	1.13 (0.91 to 1.40)	0.258	0.89 (0.74 to 1.07)	0.225	1.01 (0.80 to 1.27)	0.940	0.78 (0.59 to 1.02)	0.072
Rural	1.13 (0.93 to 1.37)	0.232	**0.75 (0.62 to 0.91)**	**0.003**	1.13 (0.96 to 1.33)	0.155	0.87 (0.71 to 1.06)	0.154	1.15 (0.87 to 1.53)	0.320
**Obesity class***										

Class I	1.01 (0.87 to 1.16)	0.952	0.89 (0.77 to 1.04)	0.141	1.01 (0.88 to 1.14)	0.942	0.91 (0.78 to 1.07)	0.249	0.94 (0.77 to 1.16)	0.579
Class II	0.86 (0.51 to 1.45)	0.564	0.92 (0.57 to 1.48)	0.729	1.09 (0.73 to 1.62)	0.681	1.06 (0.65 to 1.73)	0.825	0.84 (0.44 to 1.61)	0.600
Class III	**0.08 (0.01 to 0.68)**	**0.020**	2.82 (0.53 to 15.00)	0.224	0.46 (0.09 to 2.33)	0.350	0.49 (0.17 to 1.42)	0.188	3.20 (0.72 to 14.18)	0.126
**Drinking status**										
Yes	0.98 (0.82 to 1.18)	0.856	0.96 (0.81 to 1.15)	0.680	0.97 (0.83 to 1.13)	0.697	0.87 (0.74 to 1.03)	0.102	1.03 (0.82 to 1.30)	0.778
No	1.01 (0.81 to 1.26)	0.945	0.81 (0.65 to 1.00)	0.053	1.10 (0.91 to 1.32)	0.327	1.03 (0.81 to 1.32)	0.811	0.81 (0.59 to 1.11)	0.193
**Binge drinking**										
Yes	1.05 (0.72 to 1.53)	0.800	1.12 (0.75 to 1.66)	0.575	0.98 (0.70 to 1.37)	0.895	0.75 (0.50 to 1.12)	0.155	1.29 (0.75 to 2.21)	0.356
No	0.98 (0.85 to 1.13)	0.759	0.88 (0.76 to 1.02)	0.080	1.02 (0.90 to 1.16)	0.744	0.96 (0.82 to 1.13)	0.628	0.92 (0.74 to 1.13)	0.416
**Smoking status**										
Smoker	1.14 (0.85 to 1.52)	0.382	0.83 (0.62 to 1.11)	0.214	1.10 (0.85 to 1.41)	0.472	0.79 (0.57 to 1.10)	0.163	1.08 (0.69 to 1.69)	0.738
Non-smoker	0.94 (0.80 to 1.10)	0.423	0.94 (0.80 to 1.10)	0.441	0.99 (0.87 to 1.14)	0.915	0.97 (0.82 to 1.15)	0.753	0.92 (0.74 to 1.15)	0.477
**Level of education**										
High school or lower education	1.08 (0.91 to 1.30)	0.386	0.93 (0.77 to 1.12)	0.424	1.07 (0.90 to 1.28)	0.430	0.86 (0.68 to 1.10)	0.224	1.22 (0.89 to 1.67)	0.225
College or higher education	0.94 (0.76 to 1.16)	0.562	0.95 (0.77 to 1.17)	0.611	1.04 (0.88 to 1.22)	0.670	1.00 (0.84 to 1.20)	0.977	0.82 (0.64 to 1.03)	0.092
**Household income**										
Lowest and second quartile	0.99 (0.79 to 1.24)	0.899	0.93 (0.74 to 1.16)	0.503	0.99 (0.82 to 1.20)	0.911	1.05 (0.82 to 1.35)	0.688	0.97 (0.70 to 1.34)	0.847

Third and highest quartile	0.99 (0.83 to 1.18)	0.900	0.90 (0.76 to 1.08)	0.258	1.03 (0.89 to 1.21)	0.666	0.89 (0.75 to 1.07)	0.205	0.94 (0.76 to 1.18)	0.599

Abbreviations: BMI, body mass index; CI, confidence interval; MHO, metabolically healthy obese; MUHO, metabolically unhealthy obese; KNHANES, Korea National Health and Nutrition Examination Survey;

*Obesity classes were defined based on body mass index (BMI) according to the Korean Society for the Study of Obesity guidelines: class I (25.0–29.9 kg/m²), class II (30.0–34.9 kg/m²), and class III (≥ 35.0 kg/m²).

Table S2. Weighted odds ratios for metabolically healthy and unhealthy obesity stratified by pandemic period.

Variables	Before the pandemic (2007–2019)			During the pandemic (2020–2021)			Overall (2007–2021)		
	MHO	MUHO	p-value	MHO	MUHO	p-value	MHO	MUHO	p-value
	wOR (95% CI)	wOR (95% CI)		wOR (95% CI)	wOR (95% CI)		wOR (95% CI)	wOR (95% CI)	
**Sex**									
Female	1.00 (ref)	1.00 (ref)		1.00 (ref)	1.00 (ref)		1.00 (ref)	1.00 (ref)	
Male	0.98 (0.90 to 1.07)	1.02 (0.94 to 1.11)	0.631	0.96 (0.83 to 1.11)	1.04 (0.90 to 1.21)	0.558	0.98 (0.89 to 1.08)	1.02 (0.93 to 1.13)	0.655
**Age**									
19–39 years	1.00 (ref)	1.00 (ref)		1.00 (ref)	1.00 (ref)		1.00 (ref)	1.00 (ref)	
40–59 years	**0.44 (0.40 to 0.48)**	**2.30 (2.10 to 2.52)**	**<0.001**	**0.39 (0.33 to 0.45)**	**2.58 (2.22 to 3.00)**	**<0.001**	**0.40 (0.37 to 0.45)**	**2.48 (2.24 to 2.74)**	**<0.001**
≥60 years	**0.15 (0.13 to 0.17)**	**6.68 (5.85 to 7.63)**	**<0.001**	**0.13 (0.11 to 0.16)**	**7.82 (6.45 to 9.49)**	**<0.001**	**0.14 (0.12 to 0.16)**	**7.10 (6.18 to 8.15)**	**<0.001**
**Region of residence**									
Urban	1.00 (ref)	1.00 (ref)		1.00 (ref)	1.00 (ref)		1.00 (ref)	1.00 (ref)	
Rural	**0.91 (0.83 to 1.00)**	**1.10 (1.00 to 1.20)**	**0.047**	**0.85 (0.74 to 0.98)**	**1.17 (1.02 to 1.34)**	**0.024**	**0.88 (0.80 to 0.97)**	**1.13 (1.03 to 1.25)**	**0.010**
**Obesity class***									
Class I	1.00 (ref)	1.00 (ref)		1.00 (ref)	1.00 (ref)		1.00 (ref)	1.00 (ref)	
Class II	**0.59 (0.50 to 0.70)**	**1.70 (1.44 to 2.00)**	**<0.001**	**0.54 (0.42 to 0.69)**	**1.86 (1.45 to 2.38)**	**<0.001**	**0.56 (0.47 to 0.67)**	**1.77 (1.49 to 2.12)**	**<0.001**
Class III	**0.36 (0.23 to 0.57)**	**2.77 (1.76 to 4.37)**	**<0.001**	**0.48 (0.27 to 0.84)**	**2.08 (1.19 to 3.66)**	**0.011**	**0.44 (0.28 to 0.70)**	**2.26 (1.42 to 3.59)**	**0.001**

**Drinking status**									
No	1.00 (ref)	1.00 (ref)		1.00 (ref)	1.00 (ref)		1.00 (ref)	1.00 (ref)	
Yes	**1.34 (1.22 to 1.46)**	**0.75 (0.69 to 0.82)**	**<0.001**	**1.39 (1.22 to 1.58)**	**0.72 (0.63 to 0.82)**	**<0.001**	**1.36 (1.24 to 1.49)**	**0.74 (0.67 to 0.81)**	**<0.001**
**Binge drinking**									
No	1.00 (ref)	1.00 (ref)		1.00 (ref)	1.00 (ref)		1.00 (ref)	1.00 (ref)	
Yes	**0.78 (0.69 to 0.89)**	**1.28 (1.13 to 1.46)**	**<0.001**	**0.76 (0.62 to 0.93)**	**1.32 (1.07 to 1.63)**	**0.008**	**0.78 (0.68 to 0.90)**	**1.28 (1.12 to 1.47)**	**<0.001**
**Smoking status**									
No	1.00 (ref)	1.00 (ref)		1.00 (ref)	1.00 (ref)		1.00 (ref)	1.00 (ref)	
Yes	0.91 (0.82 to 1.01)	1.10 (0.99 to 1.23)	0.066	0.93 (0.79 to 1.10)	1.07 (0.91 to 1.27)	0.416	0.92 (0.83 to 1.03)	1.08 (0.97 to 1.21)	0.162
**Level of education**									
College or higher education	1.00 (ref)	1.00 (ref)		1.00 (ref)	1.00 (ref)		1.00 (ref)	1.00 (ref)	
High school or lower education	**0.47 (0.43 to 0.52)**	**2.11 (1.94 to 2.30)**	**<0.001**	**0.44 (0.39 to 0.50)**	**2.28 (2.00 to 2.60)**	**<0.001**	**0.46 (0.42 to 0.50)**	**2.20 (2.01 to 2.40)**	**<0.001**
**Household income**									
Third and highest quartile	1.00 (ref)	1.00 (ref)		1.00 (ref)	1.00 (ref)		1.00 (ref)	1.00 (ref)	
Lowest and second quartile	**0.70 (0.64 to 0.77)**	**1.43 (1.30 to 1.56)**	**<0.001**	**0.64 (0.56 to 0.73)**	**1.57 (1.38 to 1.79)**	**<0.001**	**0.66 (0.60 to 0.72)**	**1.52 (1.39 to 1.67)**	**<0.001**

Abbreviations: BMI, body mass index; CI, confidence interval; MHO, metabolically healthy obese; MUHO, metabolically unhealthy obese; KNHANES, Korea National Health and Nutrition Examination Survey;

*Obesity classes were defined based on body mass index (BMI) according to the Korean Society for the Study of Obesity guidelines: class I (25.0–29.9 kg/m²), class II (30.0–34.9 kg/m²), and class III (≥ 35.0 kg/m²).

Supplementary Figures

Figure S1. Study population flowchart

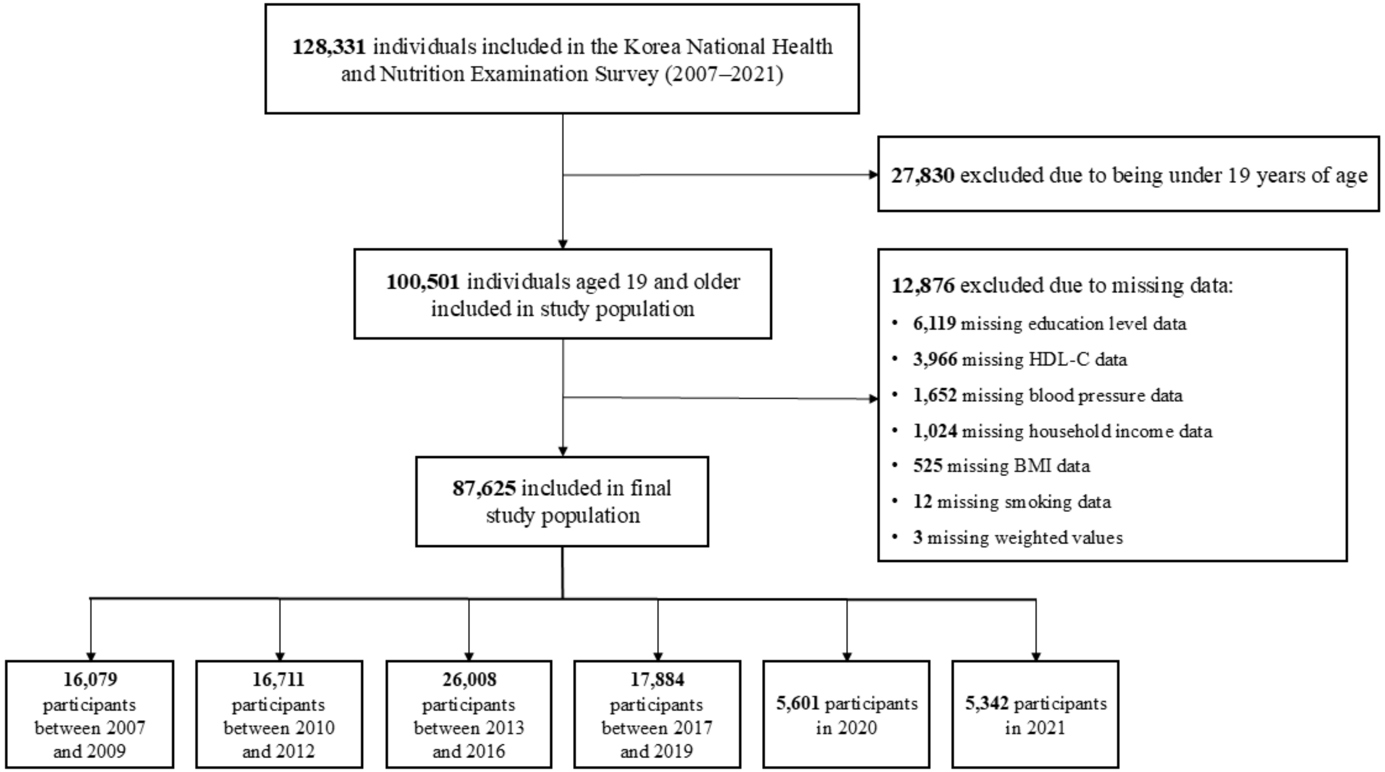

Abbreviations: BMI, body mass index; HDL-C: high-density lipoprotein cholesterol
